# Early-Life Exposure to Perfluoroalkyl Substances (PFAS) and Child Language and Communication Development: A Systematic Review

**DOI:** 10.3390/ijerph20247170

**Published:** 2023-12-12

**Authors:** Charlotte Stübner, Christel Nielsen, Kristina Jakobsson, Christopher Gillberg, Carmela Miniscalco

**Affiliations:** 1 Gillberg Neuropsychiatry Centre, Institute of Neuroscience and Physiology, Sahlgrenska Academy, University of Gothenburg, 413 90 Gothenburg, Sweden; christopher.gillberg@gnc.gu.se (C.G.); carmela.miniscalco@neuro.gu.se (C.M.); 2 Department of Pediatric Speech and Language Pathology, Queen Silvia Children’s Hospital, Sahlgrenska University Hospital, 416 50 Gothenburg, Sweden; 3 Department of Laboratory Medicine, Division of Occupational and Environmental Medicine, Lund University, 223 81 Lund, Sweden; christel.nielsen@med.lu.se; 4 Clinical Pharmacology, Pharmacy and Environmental Medicine, Department of Public Health, University of Southern Denmark, 5000 Odense, Denmark; 5 School of Public Health and Community Medicine, Sahlgrenska Academy, University of Gothenburg, 413 90 Gothenburg, Sweden; kristina.jakobsson@amm.gu.se; 6 Occupational and Environmental Medicine, Sahlgrenska University Hospital, 413 90 Gothenburg, Sweden; 7 Department of Child and Adolescent Neuropsychiatry Unit, Queen Silvia Children’s Hospital, Sahlgrenska University Hospital, 416 50 Gothenburg, Sweden

**Keywords:** child language development, developmental language disorder, environmental contaminants, perfluoroalkyl substances, prenatal exposure, postnatal exposure

## Abstract

Language development starts during the fetal period when the brain is sensitive to endocrine disruptions from environmental contaminants. This systematic review aims to systematically summarize the existing literature on early-life exposure to PFAS and children’s language and communication development, which is an indicator of neurocognitive development. A structured literature search was conducted using three databases, PubMed, Scopus, and CINAHL, last updated in April 2023. The population was defined as children and young adults. PFAS exposure was assessed pre- or postnatally. The outcome was defined as a language and communication ability assessed with validated instruments, parental self-reports, or clinical language disorder diagnoses. In total, 15 studies were identified for subsequent analyses. Thirteen were performed in background-exposed populations and two in highly exposed populations. There were some indications of potential adverse effects; however, these were not consistent across child sex, age of assessment, or PFAS exposure levels. No systematic effect of early-life PFAS exposure on language and communication development was found. These inconclusive findings may partly be explained by the use of general test instruments with limited validity as to children’s language and communication development. Further studies over a wider exposure range using specific language test instruments are needed.

## 1. Introduction

Children’s language, cognitive, and motor development follows universal milestones and develops in parallel [1]. Language development starts during the fetal period [2,3,4] and continues through preschool age [5] and well into adolescence [6]. 

Developmental language disorder (DLD) is one of the most common developmental disorders and affects 7–8% of all children [7,8]. The aetiology of DLD is multifactorial, involving both genetic and environmental risk factors [9,10]. Children with DLD have significant difficulties in one or more areas of spoken or written language, comprehension, and communication, including social interactions. If delayed or disordered language and communication persist at 5 years of age, the condition is considered permanent [9,11], and it is likely to be a risk factor for adverse social and educational performance [12]. Language and communication development is an important part of children’s general development [7,13,14,15]. Late language development or DLD is subsequently associated with other neurodevelopmental disorders and co-occurs with attention-deficit/hyperactivity disorder (ADHD), autism, and intellectual developmental disabilities [14,16,17]. 

Many hormones affect the development of the fetal brain [18], and prenatal language development is sensitive to the influence of sex hormones [19,20,21]. Early-life exposure to endocrine-disrupting chemicals may, thus, harm the developing brain [22,23,24,25,26]. Adverse effects can occur if exogenous substances pass across the placenta and reach fetal circulation or if they interfere with normal placenta functioning [24]. Exposure to endocrine-disrupting environmental contaminants such as polychlorinated biphenyls, lead, methylmercury, polybrominated diphenyl ethers, and organophosphates have been associated with the impaired development of both receptive (i.e., understanding of words and sentences) and expressive language (i.e., decreased mean length of utterances) [27].

A group of endocrine-disrupting contaminants that are widely spread in the environment and for which human exposure is ubiquitous are perfluoroalkyl substances (PFAS) [28,29]. PFASs are synthetically produced chemicals that are extremely stable and accumulate in humans [30,31,32,33,34]. PFAS transfers effectively from mother to child during pregnancy and lactation [32,33,34,35,36] and may interfere with thyroid functions that are essential for brain development [36]. 

Animal studies have indicated developmental toxicity as a sensitive target after prenatal PFAS exposure [37]. While a few epidemiological studies have reported adverse associations between PFAS serum levels and general neurodevelopment [36,38,39], the overall evidence is inconsistent [37]. These previous studies have focused on the potential impact of PFAS on children’s general development, but there has been a lack of studies on the impact of children’s language and communication, which is an important indicator of neurocognitive development. Therefore, we performed a systematic review of the existing literature on pre- and postnatal exposure to PFAS with a focus on children’s language and communication development.

## 2. Materials and Methods

We performed a systematic review according to the Preferred Reporting Items for Systematic Review and Meta-Analysis (PRISMA) model [40] and the Swedish Agency for Health Technology Assessment and Assessment of Social Services (2017) [41]. 

We preregistered the study protocol at the Open Science frameworks on 29 October 2020, https://osf.io/y45ez/.

### 2.1. Eligibility Criteria and Research Definition

We applied the Population–Exposure–Comparison–Outcome model (PECO) [42] to define our research question. The population was defined as children and young adults aged 0–21 years. Exposure was assessed as measured PFAS concentrations or as residential history in a highly exposed population. To this, we added outcomes for language and communication ability based on the use of valid test instruments, parental self-reports, or clinical diagnoses. We included original peer-reviewed scientific studies published in the English language.

### 2.2. Information Sources and Search Methods

A stepwise literature search was performed with the help of two trained librarians at Gothenburg University Library to determine search concepts in the following three areas: (1) language and communication, including stuttering, voice, and swallowing, (2) neurodevelopmental ability problems where an assessment of language and communication abilities may be included and (3) PFAS and terms related to PFAS substances. The first search in PubMed, Scopus, and CINAHL was performed by the librarians on 16 June 2020. The specification for all databases and search concepts is available in a Supplementary File (see Supplementary File S1). An updated search to capture recently published papers was performed in April 2023, and five new records were added. During a pre-search, we found no study with language or communication as the primary study outcome using specific language test instruments. Therefore, the broader search concept was used to include proxy information about language and communication domains through instruments assessing children’s general development. Moreover, reviews and systematic reviews were checked for references that might be of interest.

### 2.3. Study Selection

The relevance assessment of record titles and abstracts that included PFAS as the exposure was first conducted by two speech and language pathologists independently, with a pre-specified intention to “include rather than exclude”. For inclusion, we required an assessment of children’s language and communication and an assessment of PFAS. In the next step, an epidemiologist and a researcher in environmental medicine scrutinized the titles and abstracts that were included to assess whether the exposure assessments and study designs seemed to be relevant.

### 2.4. Data Extraction

We extracted the following metadata from the articles: the author, year, title, continent, country or state, possible connection to other screened articles, aim, population, study design, the name of study or study cohort, ages, counts, gender distribution, and the developmental domains assessed (e.g., cognitive, motor, language, and communication). In addition, information on which test instrument was used for the assessment of language and communication and if this assessment was comprehensive or only a part of broader cognitive testing was extracted. We noted reported PFAS substances, sampling periods (e.g., gestational week and child age), and matrix (whole blood, serum, plasma, or breast milk) or modeled exposure. We focused on the legacy PFAS perfluorohexane sulfonic acid (PFHxS), perfluorooctanoic acid (PFOA), perfluorooctane sulfonic acid (PFOS), and perfluorononanoic acid (PFNA).

An additional search in the Supplementary Material was also performed to obtain the information needed for decisions on inclusion and quality assessment. The validation of data followed the extraction of data, and during this process, decisions for inclusion and exclusion were made. Specific information on outcomes related to language ability was extracted from the main and supplementary text, tables, and figures. The summary measures of the results, i.e., medians, means, risk ratio, odds ratio, hazard ratio, and standard deviation, were extracted from the main and Supplementary Material. 

### 2.5. Risk of Bias Assessment

The assessment was completed using the Critical Appraisal Skills Programme and checklist for cohorts [43]. When necessary, additional information was added from supplements. All questions in the checklist were answered, but we primarily focused on a quality assessment concerning the following questions:

- “Were the participants recruited acceptably?”
- “Was the exposure accurately measured to minimize bias?”
- “Was the outcome accurately measured to minimize bias?”
- “Have the authors identified all the important confounding factors?”
- “Have they taken into account the confounding factors in the design and/or analysis?”

## 3. Results

### 3.1. Study Selection 

**Figure 1:**
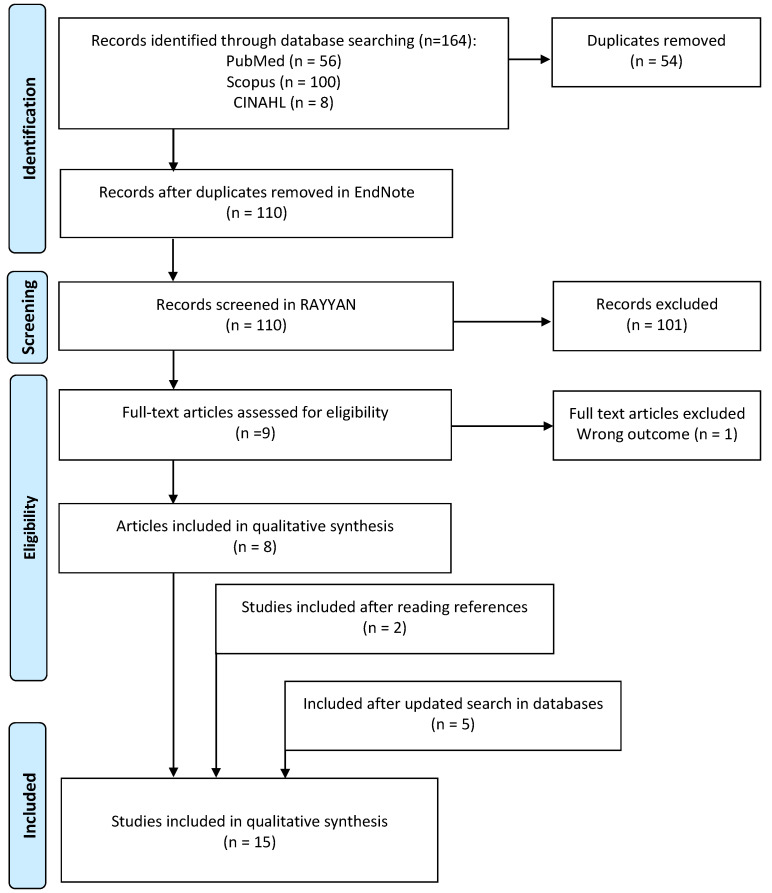
PRISMA flow chart diagram of study selection.

The final study sample consisted of 15 studies (Table 1) published between 2013 and 2023. The initial literature search identified 164 records, and after the removal of 54 duplicates via EndNote 20, [44], the remaining 110 titles and abstracts were imported to the Rayyan review app [45]. After screening titles and abstracts, there were nine articles imported to EndNote in full text. We performed full-text screening for relevance. During this process, one article was excluded because the outcome was irrelevant to the research question. After searching the reference lists of the included articles, we identified and included two additional studies. An updated literature search in April 2023 added five articles that had been published after the initial search. This process is shown in the PRISMA flow diagram (Figure 1).

### 3.2. Study Characteristics

Twelve studies were population-based birth cohorts (Table 1), all with maternal pregnancy or cord blood samples (Table 2). In addition, two studies collected childhood serum samples. 

Three studies were initiated in response to a point source of exposure. One was a birth cohort that was part of a program collecting data on contaminants and health outcomes after the collapse of the World Trade Center [46]. Two studies were part of large research programs assessing health effects after exposure to contaminated drinking water. The first was performed within the C8 Study Panel framework with exposure dominated by PFOA [47]. The second was part of the Ronneby PFAS Research Program, with exposure dominated by PFOS and PFHxS [48,49]. Both studies included prenatal exposure assessments.

Thirteen studies focused on children’s general cognitive development and included verbal subscales (Table 3), whereas two studies had a specific focus on language and communication development (i.e., Jeddy et al., 2017 and Stübner et al., 2023) [49,50]. 

The publications were from three continents, including six from Asia (China, Japan, Taiwan), five from Europe (Denmark, England, Norway, Spain, Sweden), and four from the U.S. The number of participants ranged from 120 to 11,895. Two publications reported data from the same cohort at different ages of follow-up [51,52].

One study included only girls [50]. In the remaining studies, there was an even distribution between the sexes. 

**Table 1:** General characteristics of studies included in the systematic review.

Author Year	Geographical Area	Cohort	Enrollment Year	Age ^1^ at Outcome Assessment	Number of Participants ^2^	Sex Distribution boys, %
Carrizosa et al., 2021 [53]	Europe, Spain	INfancia y Medio Ambiente (Environment and Childhood) (INMA)	2003–2008	14 months 4–5 years	1131 1192	51
Chen et al., 2013 [54]	Asia, Taiwan	Taiwan Birth Panel Study (TBPS)	2004–2005	2 years	239	55
Goudarzi et al., 2016 [55]	Asia, Japan	The Hokkaido Study on Environment and Children’s health	2002–2005	6 months 18 months	173 133	48 50
Harris et al., 2018 [56]	USA, Boston	Project Viva	1999–2002	3 years 7 years	986 865	52 52
Jeddy et al., 2017 [50]	Europe, England	The Avon Longitudinal Study of Parents and Children (ALSPAC)	1991–1992	15 months 38 months	432	0
Liew et al., 2018 [57]	Europe, Denmark	Lifestyle During Pregnancy Study nested within the Danish National Birth Cohort	2003–2008	5 years	1592	52
Luo et al., 2022 [51]	Asia, China	Shanghai Birth Cohort (SBC)	2013–2016	2 years	2257	51
Oh et al., 2022 [58]	Asia, Japan	Hamamutsu Birth Cohort (HBC Study)	2007–2012	Eight times between 4 to 40 months	~550	52
Skogheim et al., 2020 [59]	Europe, Norway	ADHD Study nested within the Norwegian Mother, Father, and Child Cohort Study	1999–2008	3.5 years	944	51
Spratlen et al., 2020 [46]	USA, New York	A cohort with prenatal exposure to the World Trade Center disaster	2001–2002	1 year 2 years 3 years 4 years 6 years	156 157 127 124 110	50
Stein et al., 2013 [47]	USA, West Virginia, and Ohio	A child subcohort from the C8 Health Project	2005–2010	6–12 years (mean age 10 years)	320	47
Stübner et al., 2023 [49]	Europe, Sweden	Register-based cohort study in Ronneby, Sweden	1998–2013	3.2–4.6 years	11,895	54
Vuong et al., 2019 [60]	USA, Ohio	Health Outcomes and Measures of the Environment (HOME)	2003–2006	8 years	221	45
Wang et al., 2015 [38]	Asia, Taiwan	The Taiwan Maternal and Infant Cohort Study	2000–2001	5 years 8 years	120 120	52 ^3^ 50
Wang et al., 2023 [52]	Asia, China	Shanghai Birth Cohort	2013–2016	4 years	2031	52

^1^ For studies with repeated outcome assessments, the number of participants at each assessment is given. ^2^ No numbers for sex at each age are reported. ^3^ Only information about means for the first speech and language pathologist visit was reported.

**Table 2:** Exposure characteristics, including the PFAS sampling period, matrix, and exposure levels for PFOA, PFOS PFHxS, and PFNA.

Author Year	PFAS Included in Statistical Analysis	Sampling Period	Matrix	PFOA (ng/mL)	PFOS (ng/mL)	PFHxS (ng/mL)	PFNA (ng/mL)
Carrizosa et al., 2021 [53]	PFOA, PFOS, PFHxS, PFNA	Trimester 1	Maternal plasma	Median (IQR) 2.4 (1.6, 3.3)	Median (IQR) 6.1 (4.4, 7.8)	Median (IQR) 0.6 (0.4, 0.8)	Median (IQR) 0.7 (0.5, 0.9)
Chen et al., 2013 [54]	PFOA, PFOS	Delivery	Plasma from cord blood	Mean (SD) 2.5 (2.6)	Mean (SD) 7.0 (5.8)	-	-
Goudarzi et al., 2016 [55]	PFOA, PFOS	After 2nd trimester	Maternal serum	Median (IQR) 1.2 (0.8, 1.7)	Median (IQR) 5.7 (4.4, 7.4)	-	-
Harris et al., 2018 [56]	PFOA, PFOS, PFHxS, PFNA, PFDeA, MeFOSAA, EtFOSAA,	Trimester 1, 2 Postnatal	Maternal plasma Child plasma midchildhood at Median age 7.7 years (6.6–10.9 year)	Maternal Median (IQR) 5.6 (4.1, 7.7) Childhood Median (IQR) 4.4 (3.1, 6.0)	Maternal Median (IQR) 24.9 (18.4, 34.4) Childhood Median (IQR) 6.2 (4.2, 9.7)	Maternal Median (IQR) 2.4 (1.6, 3.7) Childhood Median (IQR) 1.9 (1.2, 3.4)	Maternal Median (IQR) 0.6 (0.5, 0.9) Childhood Median (IQR) 1.5 (1.1, 2.3)
Jeddy et al., 2017 [50]	PFOA, PFOS, PFNA, PFHxS,	Median week 15	Maternal serum	Median (IQR) 3.7 (2.8, 4.8)	Median (IQR) 19.8 (15.0, 24.95)	Median (IQR) 1.6 (1.2, 2.2)	Median (IQR) 0.5 (0.4, 0.7)
Liew et al., 2018 [57]	PFOA, PFOS, PFHxS, PFNA, PFHpS, PFDA, PFOSA	Trimester 1	Maternal plasma	Median (IQR) 4.3 (3.2, 5.5)	Median (IQR) 28.1 (21.6, 35.8)	Median (IQR) 1.1 (0.8, 1.4)	Median (IQR) 0.46 (0.36, 0.57)
Luo et al., 2022 [51]	PFOA, PFOS, PFHxS, PFNA PFHpA, PFDeA, PFUnDA, PFDoA, PFBS	Trimester 1	Maternal plasma	Median (IQR) 11.90 (9.30, 15.20)	Median (IQR) 9.56 (6.65, 13.87)	Median (IQR) 0.54 (0.42, 0.68)	Median (IQR) 1.74 (1.25, 2.39)
Oh et al., 2022 [58]	PFOA, PFOS	Postnatal	Serum from cord blood	Median (IQR) 1.2 (0.8, 1.8)	Median (IQR) 1.2 (0.9, 1.7)	-	-
Skogheim et al., 2020 [59]	PFOA, PFOS, PFHxS, PFNA, PFHpS, PFDA, PFUnDA	Trimester 2	Maternal plasma	Median (IQR) 2.5 (1.8, 3.2)	Median (IQR) 11.5 (8.8, 14.8)	Median (IQR) 0.7 (0.5, 0.9)	Median (IQR) 0.4 (0.3, 0.5)
Spratlen et al., 2020 [46]	PFOA, PFOS, PFHxS, PFNA	Delivery	Serum from cord blood and maternal blood day after delivery	Geometric Mean (Range) Cord: 2.31 (0.18, 8.14) Maternal 2.42 (0.88, 5.06)	Geometric Mean (Range) Cord: 6.03 (1.05, 33.7) Maternal: 11.9 (2.90, 30.9)	Geometric Mean (Range) Cord: 0.67 (0.08, 15.8) Maternal: 0.94 (0.35, 3.20)	Geometric Mean (Range) Cord: 0.43 (<LOQ, 10.3) Maternal: 0.43 (<LOQ, 10.3)
Stein et al., 2013 [47]	PFOA	Estimated prenatal Postnatal	Modeled prenatal exposure Serum collected postnatally at mean age 5.7 years	Estimated in utero mean (SD) 115.9 (164.6) Childhood mean (SD) 91.1 (139.8)	Measured childhood mean (SD) 21.1 (13.3)	Measured childhood mean (SD) 9.8 (14.1)	Measured childhood mean (SD) 1.9 (1.1)
Stübner et al., 2023 [49]	PFOA, PFOS, PFHxS	Estimated early life	Maternal residential history, i.e., with or without highly PFAS-contaminated drinking water, during the five-year period before childbirth was used as a proxy for early-life exposure.	High exposure Median 9 ng/mL Intermediate Median 3 ng/mL Background Median 2 ng/mL	High exposure Median 169 ng/mL Intermediate Median 48 ng/mL Background Median 4 ng/mL	High exposure Median129 ng/mL Intermediate Median 40 ng/mL Background Median 0.8 ng/mL	-
Vuong et al., 2019 [60]	PFOA, PFOS, PFHxS, PFNA	Trimester 2 or 3 Postnatal	Maternal serum, gestation week 16 ± 3, or 26, or within 24 h of parturition. If more than one measure were taken, an average of were used. Child serum, 3 and 8 years of age	Prenatal 2nd Tertile <3.9 4.0, 6.3 ≥ 6.4 3 years 2nd Tertile <4.1 4.1, 6.7 ≥ 6.8 8 years 2nd Tertile <2.0 2.0, 2.8 ≥ 2.9	Prenatal 2nd Tertile <10.0 10.0, 15.6 ≥ 15.7 3 years 2nd Tertile <5.0 5.0, 7.9 ≥ 8.0 8 years 2nd Tertile <3.0 3.0, 4.5 ≥ 4.6	Prenatal 2nd Tertile <0.9 0.9, 1.8 ≥ 1.9 3 years 2nd Tertile <1.2 1.2, 2.4 ≥ 2.5 8 years 2nd Tertile <1.0 1.0, 1.5 ≥ 1.6	Prenatal 2ndTertile <0.7 0.7, 0.9 ≥ 1.0 3 years 2nd Tertile <1.0 1.0, 1.5 ≥ 1.6 8 years 2nd Tertile <0.6 0.6, 0.8 ≥ 0.9
Wang et al., 2015 [38]	PFOA, PFOS, PFHxS, PFNA, PFDeA, PFUnDA, PFDoDA	Trimester 3	Maternal serum	5 years Median (IQR) 2.5 (1.5, 3.4) Geometric mean (95% CI) 2.0 (1.8, 2.3) 8 years Median (IQR) 2.5 (1.5, 3.3) Geometric mean (95% CI) 2.0 (1.7, 2.3)	5 years Median (IQR) 13.3 (9.8, 17.5) Geometric mean (95% CI) 11.9 (10.4–13.6) 8 years Median (IQR) 12.3 (9.5, 16.3) Geometric mean (95% CI) 11.5 (10.2, 13.1)	5 years Median (IQR) 0.7 (0.1, 1.1) Geometric mean (95% CI) 0.4 (0.3, 0.6) 8 years Median (IQR) 0.7 (0.1, 1.1) Geometric mean (95% CI) 0.5 (0.4, 0.6)	5 years Median (IQR) 1.6 (0.8, 2.4) Geometric mean (95% CI) 1.4 (1.2, 1.7) 8 years Median (IQR) 1.4 (0.8, 2.3) Geometric mean (95% CI) 1.3 (1.1, 1.6)
Wang et al., 2023 [52]	PFOA, PFOS, PFHxS, PFNA, PFHpA PFDeA, PFUnDA, PFDoA, PFBS	Trimester 1-2	Maternal plasma	Median (IQR) 13.1 (9.4, 15.5)	Median (IQR) 11.3 (6.7, 13.7)	Median (IQR) 0.6 (0.4, 0.7)	Median (IQR) 2.1 (1.3, 2.5)

Perfluoroalkyl substances (PFAS), Perfuorooctanoic acid (PFOA), Perfluorooctane sulfonic acid (PFOS), Perfluorohexane sulfonic acid (PFHxS), Perfluorononanoic acid (PFNA), Perfluoroundecanoic acid (PFUnDA),Perfluorododecanoic acid FDoDA/PFDoA), Perfluorodecanoic acid (PFDA/PFDeA), Perfuoroheptane sulfonic acid (PFHpS), Methyl perfluorooctane sulfonamido acetate (MeFOSAA), Ethyl perfluorooctane sulfonamido acetate (EtFOSAA), Perfluorooctane sulfonamide (FOSA/PFOSA), Perfluoroheptanoic acid (PFHpA), Perfluorobutane sulfonate (PFBS), Perfluorododecanoic acid (PFDoDA/PFDoA) interquartil range (IQR), confidence interval (CI), standard deviation (SD).

### 3.3. Exposure

The exposure characteristics of the studies are provided in Table 2. Fourteen studies relied on measured PFAS levels, albeit in different matrices, i.e., whole blood, serum, or plasma. The time of sampling varied between the first pregnancy trimester up to the age of 11 years. Eight studies relied on maternal exposure measurements and two on measurements in the cord blood, whereas four studies used a combination of maternal and cord/child measurements. All studies investigated exposure to PFOA, including fourteen for PFOS, eleven for PFHxS, and ten for PFNA. Three of the studies at background PFAS exposures had higher serum levels than the others [50,56,57]. Two studies investigated highly exposed populations living in areas with contaminated drinking water. One was dominated by PFOA exposure, assessed by measured child PFAS levels, and modeled maternal exposure levels during pregnancy [47]. The second study used maternal residential history and the municipal distribution of contaminated drinking water over time to construct a proxy variable of prenatal exposure and assessed its validity against measured serum levels in a smaller dataset [49]. Here, exposure to PFOS and PFHxS dominated to a lesser extent than PFOA.

### 3.4. Outcome Assessment

The child’s age at the outcome assessment ranged from 4 months to 12 years. Seven studies performed repeated outcome assessments over periods spanning from 4 months up to 8 years (Table 1).

Thirteen studies used objective instruments assessing children’s general cognitive abilities with subscales for language and communication (Table 3). One study used a valid parental questionnaire targeting children’s vocabulary, which was developed specifically to capture language and communication development [50], while another study included preschool teacher reporting [59]. Harris et al. [56] used a specific language test instrument assessing vocabulary comprehension as a language sub-domain in their 3-year assessment. Finally, one study defined the clinical outcome of a developmental language disorder according to diagnostic codes (International Classification of Disorders, tenth revision, (ICD-10) [61], set by speech and language pathologists, using data from an administrative healthcare register with the population coverage of children that had been referred to the regional speech and pathology clinic by child nurses performing routine language screening at child health care centers [49].

**Table 3:** Characteristics of outcome assessment tests or questionnaires including the domains tested and subtests including language and communication.

Acronym	Outcome Test	Developmental Domains Tested	Scoring ^1^	Subtests Including Language and Communication Domains	Validated Age Range	Used by
BSID-I	Bayley Scales of Infant and Toddler Development (Bayley, 1969, ref 1993) [62]	Developmental functioning, mental scale and motor scale	Standard scores (M = 100, SD = 15)	Receptive and expressive language presented in the mental scale	3–28 months	Carrizosa et al., 2021 [53]
BSID-II	Bayley Scales of Infant and Toddler Development -2nd Edition, (Bayley, 1993) [6]	Developmental functioning, mental developmental index [MDI]) and motor development psychomotor developmental index (PDI)	Standard scores (M = 100, SD = 15)	Receptive and expressive language presented in the mental developmental index (MDI)	1–42 months	Goudarzi et al., 2016 [55] Spratlen et al., 2020 [46]
BSID-III	Bayley Scales of Infant and Toddler Development –Third Edition, Chinese version, (Hua et al., 2019, Yue et al., 2019) [63,64]	Developmental functioning in five domains, cognitive, language, motor, social–emotional, and adaptive behavior scales.	Standard scores (M = 100, SD = 15)	Receptive and expressive communication	1–42 months	Luo et al., 2022 [51]
CDIIT	Comprehensive Developmental Inventory for Infants and Toddlers (Liao et al., 2008) [65]	Developmental areas cognition, language, motor, social, and self-care skills	Standard scores (M = 100, SD = 15)	62 items (language)	3–71 months	Chen et al., 2013 [54]
KBIT-2	Kaufman brief intelligence test–second edition (KBIT-2) (Kaufman, 2004) [66]	Cognitive ability and processing skills	Standard scores (M = 100, SD = 15), age equivalents, and percentile ranks	Verbal standard score consists of verbal knowledge, answers given by pointing to pictures. For the riddles subtest, answers given by pointing to a picture or saying a word	4–90 years	Harris et al., 2018 [56]
MSEL	Mullen scale of early learning (Mullen, 1995) [67]	Visual reception, fine motor, receptive language, and expressive language	T-scores (M = 50, SD = 10), percentile ranks, and age equivalents for each of the five domains and the single composite (M = 100, SD = 15)	Expressive language, and receptive language	0–68 months	Oh et al., 2022 [58]
MSCA	McCarthy Scales of Children’s Abilities, (Kaufman and Kaufman, 1977) [68]	Cognitive ability	Standard scores (M = 100, SD = 15)	Verbal scale including subtests of pictorial memory, word knowledge, verbal memory, verbal fluency, and opposite analogies	2–8 years	Carrizosa et al., 2021 [53]
NEPSY-II	Developmental Neuropsychological Assessment 2 edition, NEPSY-II, (Korkman et al., 2007) [69]	Neurocognitive processes, 32 subtests for use in a neuropsychological assessment with preschoolers, children, and adolescents.	Scale scores (1–19, M = 10, SD = 3)	Body part naming and identification comprehension of instructions, Oro motor sequences, phonological processing, the repetition of nonsense words, speeded naming and word generation	3–16 years	Stein et al., 2013 [47]
PPVT-III	Peabody Picture Vocabulary Test, (Dunn, 1997) [70]	Vocabulary	Raw scores to percentile ranks, age equivalents, or standard scores (M = 100, SD = 15)	Receptive vocabulary	2–90 years	Harris et al., 2018 [65]
SB-5	Stanford-Binet Intelligence Scale, Fifth Edition, (Roid, 2003) [71]	Cognitive strengths and weaknesses	Standard scores (M = 100, SD = 15) scaled scores (M = 10, SD = 3), percentile scores, confidence intervals, age equivalents	Verbal fluid reasoning, verbal knowledge, verbal quantitative reasoning, verbal visual-spatial processing, verbal working memory	2–85 year	Skogheim et al., 2020 [59]
WASI	The Wechsler Abbreviated Scale of Intelligence, (Wechsler, 1999) [72]	General intellectual ability	Standard scores (M = 100, SD = 15)	Vocabulary, similarities	6–90 year	Stein et al., 2013 [47]
WPPSI-R	Wechsler Preschool and Primary Scale of Intelligence–Revised, (Wechsler, 1990) [73]	Intellectual ability	Standard scores (M = 100, SD = 15)	Verbal scale subtests: information, comprehension, arithmetic, vocabulary, similarities, and sentences	3–7 years	Liew et al., 2018 [57] Spratlen et al., 2020 [46] Wang et al., 2015 [38] (5 year assessment)
WPPSI-IV	Wechsler Preschool and Primary Scale of Intelligence (Wechsler, 2012) [74]	Intellectual ability	Standard scores (M = 100, SD = 15)	Verbal scale subtests: information, similarities	2–7 years	Wang et al., 2023 [52]
WISC III	Wechsler Intelligence Scale for Children 3rd. edition, (Wechsler, 1991) [75]	General cognitive ability	Standard scores (M = 100, SD = 15)	The verbal scale mandatory subtests: information, similarities, arithmetic, vocabulary, and comprehension. The supplementary subtest: digit span	6–16 year	Wang et al., 2015 [38] (8 year assessment)
WISC-IV	Wechsler Intelligence Scale for Children 4th edition, (Wechsler, 2003) [76]	General cognitive ability	Standard scores (M = 100, SD = 15)	The verbal scale core subtest: similarities, vocabulary, comprehension, supplementary: information, word reasoning	6–16 year	Vuong et al., 2019 [60] Stein 2013 [47]
	**Outcome questionnaire**					
CDI	Child Development Inventory, (Ireton and Glascoe, 1995) [77]	Teacher questionnaires. Measure the child’s present development in eight areas. Include general Development Scale and items to identify parent’s concerns about child’s health and growth, vision and hearing, development, and behavior	Percentile scores, age equivalents	Expressive language	15 months– 6 years	Skogheim et al., 2020 [59]
MCDI/MB-CDI	MacArthur-Bates Communicative Development Inventories, Second Edition, (Fenson et al., 2007) [78]	Parent questionnaire. Evaluate communication in young children	Percentile scores	Communicative skills, comprehension, early vocabulary, and early grammar	8–30 months	Jeddy et al., 2017 [50]

^1.^ Higher scores indicate better performance in all tests and questionnaires. Bayley Scales of Infant and Toddler Development—2nd Edition (BSID-II), Comprehensive Developmental inventory for infants and toddlers, diagnostic test (CDIIT), Kaufman Assessment Battery for Children, Second Edition (KBIT-2), McCarthy Scales of Children’s Abilities (MSCA), Developmental NEuroPSychological Assessment, 2nd edition (NEPSY-II), Peabody Picture Vocabulary Test (PPVT-III), Stanford–Binet Intelligence Scale, Fifth Edition (SB-5), The Wechsler Abbreviated Scale of Intelligence (WASI), Wechsler Preschool and Primary Scale of Intelligence–Revised (WPPSI-R), Wechsler Intelligence Scale for Children, 3rd. edition (WISC III), Wechsler Intelligence Scale for Children, 4th edition (WISC-IV), Child Development Inventory (CDI), MacArthur-Bates Communicative Development Inventories, Second Edition (MCDI or MB-CDI), * Maternal translation occurred if primary language was not English or Chinese.

### 3.5. Association between PFAS Exposure and Language and Communication Outcomes

The findings of the included studies are summarized in Table 4. 

Among the 13 studies with background PFAS exposure levels, the majority found no statistically significant associations. Two studies reported better language ability at higher PFAS levels [53,57]. Adverse associations were reported by Luo, Chen, Yu, Huo, Wang, Nian, Tian, Xu, Zhang, and Zhang [51], and two studies reported both positive and negative effect estimates [50,58]. 

The cohort established after the World Trade Center disaster [46] exhibited PFAS levels comparable to the background exposure cohorts, albeit with a more complex situation of chemical and psychophysiological exposure for pregnant mothers. Here, no effect of PFAS exposure was seen for the verbal scales while simultaneously indicating better general neurodevelopment–mental outcomes with increasing PFAS levels.

Two studies investigated children with substantially higher PFAS exposure. A small study (*n* = 320) reported no associations between PFOA exposure and language-related test outcomes [47]. A large, registry-based study (*n* = 11,895) found an increased risk for DLD after high exposure dominated by PFOS and PFHxS, but only in girls [49]. 

Five of seven studies with repeated outcome measurements found different results between examinations [38,46,50,53,58]. Eight out of thirteen studies that reported results for several PFAS compounds showed consistent results between the compounds. 

Effect modification by sex was found in four of the eleven studies where it was investigated. Three studies had indications of an adverse risk for girls [49,55,58], while Spratlen et al. (2020) [46] found a favorable effect in girls at the age of 2 years. 

In summary, the majority of the studies did not demonstrate an association between PFAS and language and communication development, while seven reported favorable or adverse associations.

**Table 4:** Summary of study results.

Author Year	Associations between PFAS Exposure and Language Development ^1^	Overall Summary of Results ^2^		Consistency between Outcome Assessment at Different Ages	Consistency between Repeated Exposure Measurements	Consistency between PFAS Compounds	Effect Modification by Sex
		Favorable ^3^	Adverse ^4^				
Carrizosa et al., 2021 [53]	No associations at age 14 months for PFOS, PFHxS, PFOA, or PFNA. At age 4–5 years, a favorable association between PFOS and verbal subscale was also indicated for PFNA.	Yes	No	No	n.a. ^5^	No	No
Chen et al., 2013 [54]	No association at age 2 years for PFOA or PFOS.	No	No	n.a.	n.a.	Yes	n.a.
Goudarzi et al., 2016 [55]	No associations at age 6 and 18 years for PFOA or PFOS.	No	No	Yes	n.a.	Yes	Girls in highest PFOA quartile had a tendency to produce lower scores at 6 months, but not at 18 months
Harris et al., 2018 [56]	No associations at age 3 and 8 years between verbal IQ scores and PFOS, PFHxS, PFOA, or PFNA	No	No	Yes	Yes	Yes	No
Jeddy et al., 2017 [50]	At 15 months, there is a favorable association between verbal comprehension and vocabulary. At 38 months, both a favorable and adverse association for PFOS, PFHxS, PFOA, or PFNA is found.	Yes	Yes	No	n.a.	Yes	n.a.
Liew et al., 2018 [57]	No associations for PFOS, PFHxS or PFOA at age 5 years. Indication of favorable association between verbal IQ score and PFNA at age 5 years.	Yes	No	n.a.	n.a.	No	No
Luo et al., 2022 [51]	Adverse association between PFHxS and PFNA, but not PFOS and PFOA for language scores at age 2 years.	No	Yes	n.a.	n.a.	No	n.a.
Oh et al., 2022 [58]	No associations were observed except an adverse association between PFOA and receptive language at 10 months and a favorable association between PFOS and expressive language at 24 and 32 months. Longitudinal changes in scores from 4 to 40 months of age indicated a favorable association between receptive and expressive language and PFOA and PFOS.	Yes	Yes	No	n.a.	No	Effect modification with a worse outcome for girls at 10, 18 and 40 months, but not at other timepoints
Skogheim et al., 2020 [59]	No associations for PFOS, PFHxS, PFOA or PFNA at age 3.5 years.	No	No	n.a.	n.a.	Yes	No
Spratlen et al., 2020 [46]	No association between PFOS, PFHxS, PFOA or PFNA and MDI at age 1 year, but a tendency for favorable scores at age 2 and 3 years. No associations were observed for the verbal IQ score at age 4 years	No	No	No	n.a.	Yes	Stronger positive association between PFOS and MDI for girls at age 2 years.
Stein et al., 2013 [47]	No associations for PFOA at age 6–12 years.	No	No	n.a.	Yes	n.a.	No
Stübner et al., 2023 [49]	Adverse association between PFAS exposure and a clinical diagnosis of delayed language disorder in pre-school girls, but not in boys.	No	Yes	n.a.	n.a.	n.a.	Adverse effect in girls but not boys
Vuong et al., 2019 [60]	No associations for PFOS, PFHxS and PFOA and verbal comprehension at age 8 years. For PFNA, a trend for a favorable outcome was indicated.	No	No	n.a.	No	No	No
Wang et al., 2015 [38]	No associations for PFOS, PFHxS, PFOA or PFNA at age 5 years. At 8 years, no associations except an adverse association for PFNA	No	Yes	No	n.a.	No	n.a.
Wang et al., 2023 [52]	No associations for PFOS, PFHxS, PFOA or PFNA at age 4 years.	No	No	n.a.	n.a.	Yes	No

^1^ The tests used are reported in Table 3. ^2^ Confidence interval for beta estimates either below or above 0 and a confidence interval for relative risk estimates not including 1.0 or *p* < 0.05. ^3^ Better outcomes at higher PFAS levels. ^4^ Worse outcomes at higher PFAS levels. ^5^ Not applicable.

### 3.6. Quality Assessment

#### 3.6.1. Selection Bias 

We identified no evident risk of selection bias from conditioning participation on both exposure and outcome in any of the studies. The study by Chen et al. [54], excluded children with physician-diagnosed neurodevelopmental disorders at age 2 years. The study by Skogheim et al. [59], had an enriched sample (20%) of children with ADHD symptoms at age 3 years according to parental interviews, and excluded a few children with high questionnaire scores on autistic symptoms. However, as only a very small proportion of children with neurodevelopmental disorders such as autism or ADHD receive a clinical diagnosis at such an early age [79], we assume the impact to be of minor importance for the generalizability of their results.

#### 3.6.2. Information Bias

All PFAS analyses were performed in established laboratories. 

The studies that used a modeled exposure assessment provided cross-validation between modeled and measured data [47,49]. Moreover, in these studies, the exposure levels spanned over a very wide range, which reduced the risk of exposure misclassification. By contrast, analytical uncertainty has a larger relative impact at low exposure levels. 

There was a lack of information about blinding regarding exposure in three studies with objective outcome assessments [46,54,59] and in the study using parental questionnaires [50]. The remaining eleven studies with objective outcome assessments had assessors blinded to participants’ PFAS exposure levels.

Most studies relied on instruments assessing general cognitive abilities with verbal subscales. Although these tests can provide essential information about language difficulties, it is important to remember that such tests are not primarily designed to assess language ability.

#### 3.6.3. Confounding

In general, studies were adjusted for the confounders that we a priori considered to be relevant (i.e., maternal age, socioeconomic status, parity, and smoking during pregnancy), and presented a theoretical ground as to why they were included. All studies presented both unadjusted and adjusted results in the main text or the Supplementary Materials.

## 4. Discussion

This systematic review identified fifteen studies that investigated the association between early-life exposure to PFAS (PFOA, PFOS, PFHxS, and PFNA) and language and communication development. Overall, there were no consistent findings for associations between early-life exposure to PFAS and language and communication development, neither adverse nor favorable. Sex dimorphic effects were reported in some studies, albeit in different directions. 

All but two studies were performed in birth cohorts, with substantial variation concerning the timepoint for outcome assessments. The majority of studies used well-known, validated developmental or cognitive test instruments, capturing crude measures of language and speech domains as subtests within general test batteries. In marked contrast, one study relied on standardized expert diagnoses of DLD [49], thus detecting clinically important outcomes. 

One explanation for the inconsistent findings might be related to the lack of specific language test instruments for outcome assessments. Although developmental tests or cognitive tests aim to assess the intellectual capacity of children and adolescents and can provide valuable information about possible language difficulties, these tests were not primarily designed to assess language difficulties. For example, children with expressive language disorder who struggle with grammar and speech sound pronunciation problems may still have full marks on these cognitive tests. It is also possible for children with reading and writing difficulties to pass the test because the tasks are administered and answered verbally. Thus, there is a risk that estimates may have become attenuated from outcome misclassification when general instruments are used. 

Specific language test instruments are more sensitive to picking up nuances in language and communication, and they are, thus, expected to increase the validity of the outcome assessment. In addition, the use of caregiver questionnaires on language development can be a cost- and time-efficient method to capture important aspects of functional everyday communication [80,81]. This approach was used in two of the included studies [50,59]. 

An alternative strategy for outcome assessment is to use clinical developmental language disorder diagnoses to define the outcome when healthcare data are available. In Stübner et al. (2023) [49], the outcomes were defined as (a) routine language screening with validated instrument child healthcare nurses followed by referral to a speech and language pathology clinic and (b) a subsequent diagnosis set by a speech and language pathologist. An advantage of this approach is that it captures clinically relevant outcomes, but it requires routine healthcare data for the study population to be available.

Only two studies were based on cohorts with high exposure levels of PFAS, and they reported diverging findings.

The remaining 13 studies investigated background-exposed cohorts with small exposure contrasts. Thus, there is insufficient evidence to evaluate exposure–response relationships, particularly at intermediate exposure levels.

Self-selection in longitudinal cohorts is an inherent risk, and parents with better socioeconomic characteristics are more likely to enroll as well as have higher compliance over time [82]. In populations with background exposure, a higher PFAS exposure has been associated with higher parental socioeconomic status (SES) [83]. Given that language ability is positively associated with parental SES [84], the association between language ability and PFAS exposure risk is biased toward the null in background-exposed birth cohorts. In contrast, the study by Stübner et al. (2023) [49] used routinely collected administrative healthcare data to define the outcome status for the entire population. With such a study design, selection bias can be avoided. 

## 5. Conclusions

We found no consistent association between early-life exposure to PFAS and language and communication development. Most previous research was performed in populations with background levels of exposure; the timepoint of exposure and outcome assessment varied substantially, and most studies used general cognitive instruments for outcome assessment. However, the observation of an increased risk for the clinical diagnosis of developmental language disorders in highly exposed girls is of concern. Thus, research at intermediate exposure levels is needed to clarify the exposure–response relationship between early-life PFAS exposure and speech and language development. In future studies, instruments developed to assess language and communication abilities, preferably in collaboration with speech- and language pathologists with clinical expertise, should be used.

## Data Availability

This article is based on publicly available data and the sources are provided in the article.

## Supplementary Materials

The following supporting information can be downloaded at: https://www.mdpi.com/article/10.3390/ijerph20247170/s1, Supplementary material File S1: Search terms.
