# Supplementary material for: Early-Life Exposure to Perfluoroalkyl Substances (PFAS) and Child Language and Communication Development: A Systematic Review
Source: Int J Environ Res Public Health. 2023 Dec 12;20(24):7170. doi: 10.3390/ijerph20247170 (PMC10742458; doi:10.3390/ijerph20247170)
Supplement: Supplementary file 1 [file ijerph-20-07170-s001.zip › ijerph-2713210-supplementary/ijerph-2713210-conv-supplementary-material.pdf]

Supplementary file

Early-Life Exposure to Perfluoroalkyl Substances (PFAS) and Child Language and Communication Development: A Systematic Review

Charlotte Stübner, Christel Nielsen, Kristina Jakobsson, Christopher Gillberg and Carmela Miniscalco

Supplementary File S1. Search terms

PubMed

(Fluorocarbons[mesh] OR fluorocarbons[tiab] OR PFAS[tiab] OR PFASS[tiab] OR PFOA[tiab] OR PFOS[tiab] OR PFHxS[tiab] OR Perfluorinated[tiab] OR perfluorinated acid[tiab] OR perfluorinated substances[tiab] OR perfluorooctanoate[tiab] OR perfluorooctanoic acid[tiab] OR perfluorooctanesulfonic acid[tiab] OR perfluorooctane sulfonic acid[tiab] OR perfluorooctane sulfonate[tiab] OR perfluorinated alkylated substances[tiab] OR Perfluorinated alkyl substances[tiab] OR perfluoroalkyl acids[tiab] OR Perfluorohexane sulfonate[tiab] OR perfluoroalkyl substances[tiab] OR perfluorooctane sulfonic acid[Supplementary Concept] OR fluorinated organic compounds[tiab] OR PFHxS[tiab] OR Perfluorohexanesulfonic acid[tiab] OR Perfluorohexane sulfonic acid[tiab]

AND

(Language[mesh] OR language[tiab] OR languages[tiab] OR Speech[mesh] OR speech[tiab] OR speaking[tiab] OR spoken[tiab] OR Communication[mesh] OR communication[tiab] OR Verbal[tiab] OR verbal behavior[mesh] OR speech disorders[mesh] OR language disorders[mesh] OR Linguistic[tiab] OR aphasia[mesh] OR aphasia[tiab] OR alogia[tiab] OR anepia[tiab] OR landau-kleffner syndrome[tiab] OR Dysphasia[tiab] OR dysphasias[tiab] OR mutism[mesh] OR mutism[tiab] OR mutisms[tiab] OR dyslexia[mesh] OR dyslexia[tiab] OR stuttering[mesh] OR stuttering[tiab] OR iterations[tiab] OR echolalia[mesh] OR echolalia[tiab] OR anomia[mesh] OR anomia[tiab] OR anomias[tiab] OR anomic[tiab] OR dysnomia[tiab] OR dysnomias[tiab] OR dysphasia[tiab] OR reading[tiab] OR articulation disorders[mesh] OR articulation[tiab] OR articulations[tiab] OR stammering[tiab] OR speech therapy[mesh] OR SLP[tiab] OR SLT[tiab] OR apraxias[tiab] OR apraxia[tiab] OR dyspraxia[tiab] OR dyspraxias[tiab] OR agraphia[tiab] OR agraphias[tiab] OR Dysgraphia[tiab] OR Dysgraphias[tiab] OR Phonological Impairments[tiab] OR disarticulation[tiab] OR misarticulation[tiab] OR Dysarthria[tiab] OR Dysarthrosis[tiab] OR dysarthrias[tiab] OR Anarthria[tiab] OR anarthrosis[tiab] OR voice[mesh] OR voice[tiab] OR lisping[tiab] OR Hypernasality[tiab] OR Hyponasality[tiab] OR macroglossia[tiab] OR microglossia[tiab] OR deglutation disorders[mesh] OR deglutation disorder[tiab] OR deglutation disorders[tiab] OR voice disorders[mesh] OR voice[tiab])

AND

(adolescent[Mesh] OR child[Mesh] OR young adult[Mesh] OR fetus[Mesh] OR infant[Mesh] OR neonatal[tiab] OR neonate[tiab] OR neonates[tiab] OR zygote[tiab] OR zygotes[tiab] OR zygotic[tiab] OR embryo[tiab] OR embryos[tiab] OR embryonic[tiab] OR fetus[tiab] OR foetus[tiab] OR fetal[tiab] OR unborn[tiab] OR newborn[tiab] OR child[tiab] OR children[tiab] OR infant[tiab] OR infancy[tiab] OR toddler[tiab] OR toddlers[tiab] OR pre-puberty[tiab] OR pre-pubertal[tiab] OR puberty[tiab] OR pubertal[tiab] OR teen[tiab] OR teens[tiab] OR teenager[tiab] OR teenagers[tiab] OR youth[tiab] OR youths[tiab] OR adolescent[tiab] OR adolescents[tiab] OR adolescence[tiab] OR juvenile[tiab] OR juveniles[tiab] OR young[tiab] OR youngs[tiab] OR youngster[tiab] OR youngsters[tiab] OR pre-teen[tiab] OR pre-teens[tiab] OR preteen[tiab] OR preteens[tiab]

OR pre-adolescent[tiab] OR pre-adolescents[tiab] OR pre-adolescence[tiab] OR preadolescent[tiab] OR preadolescents[tiab] OR pre-adolescence[tiab] OR pediatric[tiab] OR pediatrics[tiab] OR paediatric[tiab] OR paediatrics[tiab] OR student[tiab] OR students[tiab] OR Congenital[subheading] OR congenital abnormalities[mesh] OR congenital[tiab] OR birth defect[tiab] OR birth defects[tiab] OR deformity[tiab] OR deformities[tiab])

Scopus

TITLE-ABS-KEY (Fluorocarbons OR fluorocarbon OR PFAS OR PFASS OR PFOA OR PFOS OR PFHxS OR Perfluorinated OR "perfluorinated acid" OR "perfluorinated substances" OR perfluorooctanoate OR) O "perfluorooctanoic acid" OR "perfluorooctanesulfonic acid" OR "perfluorooctane sulfonic acid" OR "perfluorooctane sulfonate" OR "perfluorinated alkylated substances" OR "Perfluorinated alkyl substances" OR "perfluoroalkyl acids" OR "Perfluorohexane sulfonate" OR "perfluoroalkyl substances" OR "perfluorooctane sulfonic acid" OR "fluorinated organic compounds" OR "Perfluorohexanesulfonic acid" OR "Perfluorohexane sulfonic acid")

AND

TITLE-ABS-KEY (Language OR languages OR Speech OR speaking OR spoken OR Communication OR Verbal OR "verbal behavior" OR "speech disorders" OR "language disorders" OR "speech disorder" OR "language disorder" OR Linguistic OR aphasia OR alogia OR anepia OR "landau-kleffner syndrome" OR Dysphasia OR dysphasias OR mutism OR mutisms OR dyslexia OR stuttering OR iterations OR echolalia OR anomia OR anomias OR anomic OR dysnomia OR dysnomias OR dysphasia OR reading OR "articulation disorders" OR articulation OR articulations OR stammering OR "speech therapy" OR SLP OR SLT OR apraxias OR apraxia OR dyspraxia OR dyspraxias OR agraphia OR agraphias OR Dysgraphia OR Dysgraphias OR "Phonological Impairments" OR disarticulation OR misarticulation OR Dysarthria OR Dysarthrosis OR dysarthrias OR Anarthria OR anarthrosis OR voice OR lisping OR Hypernasality OR Hyponasality OR macroglossia OR "deglutation disorders" OR "deglutation disorder" OR "voice disorders")

AND

TITLE-ABS-KEY(adolescent* OR child* OR "young adult" OR fetus OR infant OR neonatal OR neonate* OR zygot* OR embryo* OR foetus OR fetal OR unborn OR newborn OR infancy OR toddler* OR pre-puberty OR pre-pubertal OR puberty* OR teen* OR youth* OR juvenile* OR young* OR pre-teen* OR preteen* OR pre-adolescent* OR preadolescent* OR pediatric* OR paediatric* OR student* OR Congenital OR "congenital abnormalities" OR "birth defect" OR "birth defects" OR deformity OR deformities)

Cut and paste

((TITLE-ABS-KEY (fluorocarbons OR fluorocarbon OR pfas OR pfass OR pfoa OR pfos OR pfhxs OR perfluorinated OR "perfluorinated acid" OR "perfluorinated substances" OR perfluorooctanoate OR "perfluorooctanoic acid" OR "perfluorooctanesulfonic acid" OR "perfluorooctane sulfonic acid" OR "perfluorooctane sulfonate" OR "perfluorinated alkylated substances" OR "Perfluorinated alkyl substances" OR "perfluoroalkyl acids" OR "Perfluorohexane sulfonate" OR "perfluoroalkyl substances" OR "perfluorooctane sulfonic acid" OR "fluorinated organic compounds" OR "Perfluorohexanesulfonic acid" OR "Perfluorohexane sulfonic acid" OR))) AND (TITLE-ABS-KEY (adolescent* OR child* OR "young adult" OR fetus OR infant OR neonatal OR neonate* OR zygot* OR embryo* OR foetus OR fetal OR unborn OR newborn OR infancy OR toddler* OR pre-puberty OR pre-pubertal OR puberty* OR teen* OR youth* OR juvenile* OR young* OR pre-teen* OR preteen* OR pre-adolescent* OR preadolescent* OR pediatric* OR paediatric* OR student* OR congenital OR "congenital abnormalities" OR "birth defect" OR "birth defects" OR deformity OR deformities)) AND (TITLE-ABS-KEY (language OR languages OR speech OR speaking OR spoken OR communication OR verbal OR "verbal behavior" OR "speech disorders" OR "language disorders" OR "speech disorder" OR "language disorder" OR linguistic OR aphasia OR alogia OR anepia OR "landau-kleffner syndrome" OR dysphasia OR dysphasias OR mutism OR mutisms OR dyslexia OR stuttering OR iterations OR

echolalia OR anomia OR anomias OR anomic OR dysnomia OR dysnomias OR dysphasia OR reading OR "articulation disorders" OR articulation OR articulations OR stammering OR "speech therapy" OR slp OR slt OR apraxias OR apraxia OR dyspraxia OR dyspraxias OR agraphia OR agraphias OR dysgraphia OR dysgraphias OR "Phonological Impairments" OR disarticulation OR misarticulation OR dysarthria OR dysarthrosis OR dysarthrias OR anarthria OR anarthrosis OR voice OR lisping OR hypernasality OR hyponasality OR macroglossia OR "deglutation disorders" OR "deglutation disorder" OR "voice disorders"))

CINAHL (via översättning av PubMed-sökningen i Polyglot)

#**1** ((MH "Fluorocarbons+") OR TI fluorocarbons OR AB fluorocarbons OR TI PFAS OR AB PFAS OR TI PFASS OR AB PFASS OR TI PFOA OR AB PFOA OR TI PFOS OR AB PFOS OR TI PFHxS OR AB PFHxS OR TI Perfluorinated OR AB Perfluorinated OR TI "perfluorinated acid" OR AB "perfluorinated acid" OR TI "perfluorinated substances" OR AB "perfluorinated substances" OR TI perfluorooctanoate OR AB perfluorooctanoate OR TI "perfluorooctanoic acid" OR AB "perfluorooctanoic acid" OR TI "perfluorooctanesulfonic acid" OR AB "perfluorooctanesulfonic acid" OR TI "perfluorooctane sulfonic acid" OR AB "perfluorooctane sulfonic acid" OR TI "perfluorooctane sulfonate" OR AB "perfluorooctane sulfonate" OR TI "perfluorinated alkylated substances" OR AB "perfluorinated alkylated substances" OR TI "Perfluorinated alkyl substances" OR AB "Perfluorinated alkyl substances" OR TI "perfluoroalkyl acids" OR AB "perfluoroalkyl acids" OR TI "Perfluorohexane sulfonate" OR AB "Perfluorohexane sulfonate" OR TI "perfluoroalkyl substances" OR AB "perfluoroalkyl substances" OR "perfluorooctane sulfonic acid[Supplementary Concept]"

OR TI "fluorinated organic compounds" OR AB "fluorinated organic compounds" OR TI PFHxS OR AB PFHxS OR TI "Perfluorohexanesulfonic acid" OR AB "Perfluorohexanesulfonic acid" OR TI "Perfluorohexane sulfonic acid" OR AB "Perfluorohexane sulfonic acid")

AND

#**2** ((MH "Language+") OR TI language OR AB language OR TI languages OR AB languages OR (MH "Speech+") OR TI speech OR AB speech OR TI speaking OR AB speaking OR TI spoken OR AB spoken OR (MH "Communication+") OR TI communication OR AB communication OR TI Verbal OR AB Verbal OR (MH "verbal behavior+") OR (MH "speech disorders+") OR (MH "language disorders+") OR TI Linguistic OR AB Linguistic OR (MH "aphasia+") OR TI aphasia OR AB aphasia OR TI alogia OR AB alogia OR TI anepia OR AB anepia OR TI "landau-kleffner syndrome" OR AB "landau-kleffner syndrome" OR TI Dysphasia OR AB Dysphasia OR TI dysphasias OR AB dysphasias OR (MH "mutism+") OR TI mutism OR AB mutism OR TI mutisms OR AB mutisms OR (MH "dyslexia+") OR TI dyslexia OR AB dyslexia OR (MH "stuttering+") OR TI stuttering OR AB stuttering OR TI iterations OR AB iterations OR (MH "echolalia+") OR TI echolalia OR AB echolalia OR (MH "anomia+") OR TI anomia OR AB anomia OR TI anomias OR AB anomias OR TI anomic OR AB anomic OR TI dysnomia OR AB dysnomia OR TI dysnomias OR AB dysnomias OR TI dysphasia OR AB dysphasia OR TI reading OR AB reading OR (MH "articulation disorders+") OR TI articulation OR AB articulation OR TI articulations OR AB articulations OR TI stammering OR AB stammering OR (MH "speech therapy+") OR TI SLP OR AB SLP OR TI SLT OR AB SLT OR TI apraxias OR AB apraxias OR TI apraxia OR AB apraxia OR TI dyspraxia OR AB dyspraxia OR TI dyspraxias OR AB dyspraxias OR TI agraphia OR AB agraphia OR TI agraphias OR AB agraphias OR TI Dysgraphia OR AB Dysgraphia OR TI Dysgraphias OR AB Dysgraphias OR TI "Phonological Impairments" OR AB "Phonological Impairments" OR TI disarticulation OR AB disarticulation OR TI misarticulation OR AB misarticulation OR TI Dysarthria OR AB Dysarthria OR TI Dysarthrosis OR AB Dysarthrosis OR TI dysarthrias OR AB dysarthrias OR TI Anarthria OR AB

Anarthria OR TI anarthrosis OR AB anarthrosis OR (MH "voice+") OR TI voice OR AB voice OR TI lisping OR AB lisping OR TI Hypernasality OR AB Hypernasality OR TI Hyponasality OR AB Hyponasality OR TI macroglossia OR AB macroglossia OR TI microglossia OR AB microglossia OR (MH "deglutation disorders+") OR TI "deglutation disorder" OR AB "deglutation disorder" OR TI "deglutation disorders" OR AB "deglutation disorders" OR (MH "voice disorders+") OR TI voice OR AB voice)

AND

#**3** ((MH "adolescent+") OR (MH "child+") OR (MH "young adult+") OR (MH "fetus+") OR (MH "infant+") OR TI neonatal OR AB neonatal OR TI neonate OR AB neonate OR TI neonates OR AB neonates OR TI zygote OR AB zygote OR TI zygotes OR AB zygotes OR TI zygotic OR AB zygotic OR TI embryo OR AB embryo OR TI embryos OR AB embryos OR TI embryonic OR AB embryonic OR TI fetus OR AB fetus OR TI foetus OR AB foetus OR TI fetal OR AB fetal OR TI unborn OR AB unborn OR TI newborn OR AB newborn OR TI child OR AB child OR TI children OR AB children OR TI infant OR AB infant OR TI infancy OR AB infancy OR TI toddler OR AB toddler OR TI toddlers OR AB toddlers OR TI pre-puberty OR AB pre-puberty OR TI pre-pubertal OR AB pre-pubertal OR TI puberty OR AB puberty OR TI pubertal OR AB pubertal OR TI teen OR AB teen OR TI teens OR AB teens OR TI teenager OR AB teenager OR TI teenagers OR AB teenagers OR TI youth OR AB youth OR TI youths OR AB youths OR TI adolescent OR AB adolescent OR TI adolescents OR AB adolescents OR TI adolescence OR AB adolescence OR TI juvenile OR AB juvenile OR TI juveniles OR AB juveniles OR TI young OR AB young OR TI youngs OR AB youngs OR TI youngster OR AB youngster OR TI youngsters OR AB youngsters OR TI pre-teen OR AB pre-teen OR TI pre-teens OR AB pre-teens OR TI preteen OR AB preteen OR TI preteens OR AB preteens OR TI pre-adolescent OR AB pre-adolescent OR TI pre-adolescents OR AB pre-adolescents OR TI pre-adolescence OR AB pre-adolescence OR TI preadolescent OR AB preadolescent OR TI preadolescents OR AB preadolescents OR TI pre-adolescence OR AB pre-adolescence OR TI pediatric OR AB pediatric OR TI pediatrics OR AB pediatrics OR TI paediatric OR AB paediatric OR TI paediatrics OR AB paediatrics OR TI student OR AB student OR TI students OR AB students OR

Congenital[subheading]

OR (MH "congenital abnormalities+") OR TI congenital OR AB congenital OR TI "birth defect" OR AB "birth defect" OR TI "birth defects" OR AB "birth defects" OR TI deformity OR AB deformity OR TI deformities OR AB deformities
